# Protocol for a prospective cohort study on pre-eclampsia risk prediction in Ghana, Kenya and South Africa

**DOI:** 10.1186/s12978-025-02156-1

**Published:** 2025-10-27

**Authors:** PEARLS study collaborators, Kara Blackburn, Saima Sultana, Meghan Bohren, Robert Mahar, Lester Chinery, Alessandra Fluerent, Samuel A. Oppong, Alim Swarray-Deen, Kwame Adu-Bonsaffoh, Zahida P. Qureshi, George N. Gwako, Rosa N. Chemway, Mushi Matjila, Ayesha Osman, Basky Thilaganathan, Annie R. A. McDougall, Sue Fawcus, Ama A. Tamatey, Rachel Craik, Long Nguyen, Nicole Minckas, Julie A. Simpson, Digsu N. Koye, Jennifer Scott, A. Metin Gülmezoglu, Alfred Osoti, Joshua P. Vogel

**Affiliations:** 1 Women’s, Children’s and Adolescents’ Health Program, Burnet Institute, 85 Commercial Road, Melbourne, Melbourne, VIC 3004 Australia; 2 Gender and Women’s Health Unit, Nossal Institute for Global Health, University of Melbourne School of Population and Global Health, Melbourne, Australia; 3 University of Cape Town, Cape Town, South Africa; 4 Department of Obstetrics and Gynaecology, University of Ghana Medical School, Accra, Ghana; 5 Centre for Epidemiology and Biostatistics, Melbourne School of Population and Global Health, University of Melbourne, Melbourne, Australia; 6 Methods and Implementation Support for Clinical and Health (MISCH) Research Hub, University of Melbourne, Melbourne, Australia; 7 Concept Foundation, Geneva, Switzerland; 8 Concept Foundation, Bangkok, Thailand; 9 Department of Obstetrics and Gynaecology, University of Nairobi, Nairobi, Kenya

**Keywords:** Aspirin, Hypertensive disorders of pregnancy, Pre-eclampsia, Prognostic accuracy, Protocol, Prospective cohort, Risk screening, Sub-Saharan Africa

## Abstract

**Background:**

Low dose aspirin is recommended for the prevention of pre-eclampsia in high-risk women. As part of the formative work for the "Preventing pre-eclampsia: Evaluating AspiRin Low-dose regimens following risk Screening" (PEARLS) trial, we aim to validate and implement a pre-eclampsia risk-screening algorithm, based on a restricted-variable version of the Fetal Medicine Foundation (FMF) algorithm. In the trial phase, we will compare different daily aspirin doses (75 mg vs. 150 mg) for pre-eclampsia prevention and postpartum bleeding in high-risk women. This study protocol outlines the validation cohort for a restricted variable FMF algorithm in participating facilities in Ghana, Kenya, and South Africa.

**Methods:**

This multi-country, prognostic accuracy study using a prospective cohort will recruit 16,007 pregnant women at 51 health facilities across Kenya, Ghana and South Africa. The eligible population are pregnant women presenting for an antenatal visit from 11 weeks and 0 days to < 20 weeks’ gestation. Eligible women will be screened using a ‘restricted variable’ approach with the FMF algorithm (i.e. history and mean arterial pressure only), to identify women at high risk of preterm pre-eclampsia. This is performed via an adapted version of the Tommy’s Clinical Decision Tool. The primary objective is to estimate a preterm pre-eclampsia risk threshold that equates to a screen-positive rate of 10%. Secondary outcomes include estimation of the prognostic accuracy and predictive performance of the tool.

**Discussion:**

The study will provide critical evidence on the prognostic accuracy and predictive performance of a pre-eclampsia risk screening algorithm in sub-Saharan African settings. This study will inform the design of the PEARLS trial, as well as provide vital evidence for implementation of systematic risk screening for pre-eclampsia in African women.

**Supplementary Information:**

The online version contains supplementary material available at 10.1186/s12978-025-02156-1.

## Abstract (plain language summary)

Pre-eclampsia is a condition that affects pregnant women, causing high blood pressure as well as problems with her kidneys, organs or brain. It can also cause poor growth of her baby. To prevent pre-eclampsia, women at higher risk should be given daily, low-dose aspirin to take during pregnancy. In this paper, we describe our protocol (what we plan to do) for determining how we can best identify which women are at high risk of pre-eclampsia in Ghana, Kenya and South Africa. We will recruit women in the first half of pregnancy, and use a tool that includes questions about her medical history, as well as measuring her blood pressure and gestational age. We will follow them up to see how well the tool predicts which women will develop preterm pre-eclampsia. The study described here will inform a drug trial, called PEARLS. In that trial, we will recruit women at high risk for pre-eclampsia, and then compare two different doses of daily low-dose aspirin (150 mg or 75 mg). The trial will determine which dose is more effective and safe.

**Supplementary Information:**

The online version contains supplementary material available at 10.1186/s12978-025-02156-1.

## Background

Pre-eclampsia is defined as new onset hypertension after 20 weeks’ gestation accompanied by proteinuria or other maternal end-organ dysfunction, and is one of the leading causes of maternal death [1–3]. Globally, the prevalence of pre-eclampsia is approximately 4.6%, however pre-eclampsia is difficult to diagnose and the burden is probably underestimated [4]. Hypertensive disorders of pregnancy (HDP) cause ~ 46,000 maternal deaths each year – the majority of which are due to pre-eclampsia and eclampsia [3]. Nearly all maternal deaths (99%), including those due to pre-eclampsia, occur in low- and middle-income countries (LMICs) [1]. Preterm pre-eclampsia (pre-eclampsia with delivery < 37 weeks’ gestation) while less common, carries an increased risk of maternal death and adverse outcomes, compared to term pre-eclampsia (pre-eclampsia with delivery ≥ 37 weeks’ gestation) [5]. Many of these deaths are avoidable if pregnant women can access high-quality antenatal care throughout pregnancy. For pre-eclampsia, ideally this should include preconception care, risk screening early in pregnancy, preventive low-dose aspirin for high-risk women, and oral calcium supplementation in settings where dietary calcium intake is low [6–8]. It should also include blood pressure and proteinuria checks at each antenatal visit, appropriate use of anti-hypertensive medications, timed delivery, and magnesium sulfate to prevent eclampsia [6–9].

In 2021 WHO recommended that women at moderate-to-high risk of pre-eclampsia should receive daily low-dose aspirin, ideally commencing before 20 weeks’ gestation [8]. While WHO’s recommendations favoured a 75 mg aspirin dose, the guideline panel highlighted the uncertainty around aspirin dosing. While they acknowledged that 150 mg might be more effective (though confidence intervals are overlapping), they expressed concern about the possible risks of postpartum haemorrhage (PPH) at higher aspirin doses. The panel called for a comparative trial of 150 mg vs 75 mg aspirin to resolve this uncertainty.

The PEARLS Trial (Preventing pre-eclampsia: Evaluating AspiRin Low-dose regimens following risk Screening) addresses this question through an individually randomised, double-blind trial of pregnant women at high risk of pre-eclampsia in Ghana, Kenya and South Africa (PACTR202403785563823). India was added as a fourth site to the planned PEARLS trial, to expand the generalisability to LMICs outside of sub-Saharan Africa. PEARLS will evaluate whether 150 mg is more beneficial than 75 mg for preventing preterm pre-eclampsia (superiority hypothesis), and also assess safety, in terms of a composite outcome of PPH-related management (non-inferiority hypothesis). Before we can implement this trial, we need to determine the best way to identify women at high-risk of pre-eclampsia in LMICs.

### Risk screening for pre-eclampsia

Multiple maternal risk factors are associated with an increased risk of pre-eclampsia, including (but not limited to) history of pre-eclampsia in a previous pregnancy, chronic hypertension, race, advanced maternal age, nulliparity, multiple pregnancy and comorbidities such as obesity, diabetes and autoimmune conditions [10–12]. Identifying women who are at increased risk based on the presence of one or more of these factors (often as a checklist) has formed the basis of pre-eclampsia risk screening for decades. Preventive low-dose aspirin can be offered to these high-risk women. Many agencies internationally recommend screening for pre-eclampsia using history-based risk factors only (Table 1) [2, 14]. While this approach is relatively easy to implement, it has a low positive predictive value. That is, many women who are identified as high risk do not go on to develop pre-eclampsia. Identifying women at risk based on history alone, as recommended in the UK’s NICE 2019 guidelines, has been shown to identify only ~ 40% of women who will develop pre-eclampsia [17–19].

**Table 1:** Approaches to pre-eclampsia risk screening recommended by selected normative organisations

Agency	Who to screen	Definition of high risk	Factors used in screening
ISSHP (2018) [13]	Pregnant women (not otherwise specified)	Increased risk (depending on certain risk factors)	History-based risk factors *Where integrated into local health systems:* mean arterial pressure (MAP), PLGF, UTPI
NICE (2019) [14]	All pregnant women 11 weeks to 13 weeks 6 days gestation	High: at least 1 high risk factor, or 2 moderate risk factors	History-based risk factors only
FIGO (2019) [15]	All pregnant women before the end of the first trimester	Low (< 1 in 100) High (> 1 in 100)	*Ideal:* History-based risk factors; MAP; UTPI; PLGF *Pragmatic:* History-based risk factors; MAP
ACOG (2020) [2] USPSTF (2021) [16]	Pregnant women without hypertension or preeclampsia	High: one or more high risk-factors, or several moderate risk factors	History-based risk factors only
WHO (2021) [8]	Pregnant women (not otherwise specified)	Moderate to high risk (depending on certain risk factors)	No screening recommendation, but has a “non-exhaustive” list of risk factors

*MAP* Mean arterial pressure, *PLGF* Placental growth factor, *UTPI* Uterine artery Doppler pulsatility index

A 2024 systematic review by Tiruneh et al., which included 52 externally validated prediction models for pre-eclampsia, identified the Fetal Medicine Foundation (FMF) algorithm as the highest performer [20]. It was validated in 16 settings, and was the only included tool validated more than three times [20]. The FMF tool uses a Bayesian model combining information on history-based risk factors and gestational age, as well as mean arterial pressure (MAP), uterine artery Doppler pulsatility index (UTPI), placental growth factor (PLGF) levels and/or pregnancy-associated placental protein A (PAPP-A) levels [21, 22]. FMF advises screening at 11 to 14 weeks’ gestation. The model calculates an individual risk score, reflecting an estimated risk of the woman developing preterm pre-eclampsia (e.g. 1 in 50, or 2%).

Studies of the FMF algorithm have found it has high sensitivity in predicting preterm pre-eclampsia (< 37 weeks’ gestation), though this is lower for term pre-eclampsia [18, 22]. A pooled analysis of three cohorts in five European countries (61,174 women) used a risk cut-off of 1% or more to define high-risk women (i.e. >= 1 in 100 risk of preterm pre-eclampsia). Using this cut-off, the authors reported a sensitivity of 80.7% (95% Confidence Interval (CI) 77.0–84.0%) and a false positive rate of 10% [22]. Studies using the same cut-off have found that 10.5—14.7% of women are screen-positive (i.e. high risk) [22–24]. Comparative analyses have shown that the FMF algorithm outperforms history-based checklists for risk screening—it detected 75% of preterm pre-eclampsia, compared to NICE checklists, which detect only 39% of preterm pre-eclampsia at a similar screen-positive rate of 10% [18].

The FMF algorithm has been validated in several high-income countries, [25–27] and high and low/middle income Asian countries [17, 28]. However, blood biomarker levels may differ across populations, which can affect the proportion of women who screen-positive, and its prognostic accuracy [28]. Even in the absence of UTPI and PLGF/PAPP-A testing, using the FMF’s Bayesian algorithm with history plus MAP alone appears superior to history-based risk factor checklists [22, 29]. It has not, however, been formally validated in sub-Saharan African women. The FMF algorithm has been endorsed for clinical use by ISSHP and FIGO [13, 15]. In the UK, the Tommy’s National Centre for Maternity Improvement sponsored the development of the Tommy’s Clinical Decision Tool—a web-based, CE-marked medical device—that includes the FMF algorithm [30].

#### Knowledge gaps in pre-eclampsia risk screening

Currently, pre-eclampsia risk screening using the FMF tool has not reached routine clinical practice in many LMICs, particularly those in sub-Saharan Africa and South Asia. There are many reasons for this—the FMF tool requires special investigations (PLGF, PAPP-A testing, UTPI measurement with Doppler ultrasound) which are expensive and rarely available in limited-resource contexts. Also, many women in LMICs do not commence antenatal care early, [31] meaning that women at risk may not be identified until late in pregnancy, if at all. This is further complicated by variable availability and quality of ultrasounds in many LMICs to assess gestational age [32, 33].

The FMF tool has been developed and validated in pregnant women across multiple European and Asian countries. However, there is no published primary study on its prognostic performance in sub-Saharan African populations and settings, where the prevalence of pre-eclampsia (and pre-eclampsia associated morbidity and mortality) is considerably higher [4]. Furthermore, conditions like sickle-cell disease, malaria, HIV and TB are prevalent in LMICs, and are associated with higher risks of pre-eclampsia [34, 35]. These conditions have not been captured in prior pre-eclampsia risk screening tools, including FMF [36].

Sub-analyses of FMF studies in European settings have shown that its performance varies in different subpopulations [22]. At a 1 in 100 risk threshold, the screen-positive rate, sensitivity, and false-positive rate for Afro-Caribbean women were consistently higher than for Caucasian women (Table 2). The authors attributed this to the higher underlying risk of pre-eclampsia in these women [22].

**Table 2:** Performance of FMF tool in different populations and using different variables, assuming a risk cut-off of >= 1 in 100 (adapted from [22])

Variables	Sample Size	Population	Screen positive rate (%)	Preterm pre-eclampsia	
				Detection rate (%) (95% Confidence Interval)	False positive (%)
Maternal factors + MAP, UtA-PI, PAPP-A, PlGF	61,174	Mixed (73.5% Caucasian, 16.1% Afro-Caribbean)	14.7	80.7 (77.0–84.0)	14.1
	10,108	Afro-Caribbean only	34.0	92.3 (87.6–95.4)	32.9
Maternal factors + MAP	61,174	Mixed (73.5% Caucasian, 16.1% Afro-Caribbean)	18.3	66.7 (62.5–70.8)	17.9
	10,108	Afro-Caribbean only	58	84.7 (78.8—89.2)	45.4
Maternal factors only	61,174	Mixed (73.5% Caucasian, 16.1% Afro-Caribbean)	19.1	59.4 (55.0–63.7)	18.8
	10,108	Afro-Caribbean only	46.2	87.4 (81.9–91.5)	57.5

*MAP* Mean arterial pressure, *PAPP-A* Pregnancy-associated plasma protein-A, *PLGF* Placental growth factor, *UTPI* Uterine artery Doppler pulsatility index

### Rationale for a prospective cohort study on pre-eclampsia risk prediction in sub-Saharan Africa

The PEARLS Trial aims to resolve the question of 150 mg vs 75 mg aspirin for pre-eclampsia prevention in high-risk women (PACTR202403785563823). It will determine whether the higher dose of 150 mg/day aspirin is more effective, but also sufficiently safe when compared to the WHO-recommended 75 mg/day aspirin. This comparison is essential in making definitive policy decisions on aspirin for pre-eclampsia prevention. To do this, a systematic and accurate method of identifying high-risk women for recruitment into the trial is needed. WHO does not specifically recommend how high-risk women should be defined, though its 2021 recommendation on low-dose aspirin provides a non-exhaustive list of common conditions.

Available evidence on the FMF tool shows high sensitivity for predicting preterm pre-eclampsia, however it has not been evaluated in sub-Saharan African women or settings. Certain variables within the FMF tool are not routinely available in these settings, and women may not be able to access risk screening at 11 to 14 weeks’ gestation. We therefore planned a prospective cohort study to estimate prognostic accuracy for pre-eclampsia risk screening in these settings, using a restricted-variable version of the FMF tool. This study will also generate prevalence data for key trial outcomes and ensure that pre-eclampsia risk screening can be implemented consistently, prior to the PEARLS randomised trial of high-risk women.

## Aims, hypothesis and objectives

The aim of this prospective cohort study is to test and validate use of the ‘restricted variable’ FMF tool for predicting preterm pre-eclampsia in pregnant women in Ghana, Kenya and South Africa.

### Primary objective

- Calibration objective:
  1. To estimate a preterm pre-eclampsia risk threshold that equates to a screen-positive rate of 10%.

### Secondary objectives

- Prognostic objectives:
  1. To estimate the prognostic accuracy and predictive performance of the tool, for the outcomes: delivery with pre-eclampsia at < 37 weeks, < 32 weeks, < 34 weeks and ≥ 37 weeks’ gestation.
  2. To estimate whether prognostic accuracy varies at different gestational ages at screening (11–13, 14–16 and 17- < 20 weeks).
  3. To summarise prognostic accuracy of the tool, as compared to history-based ‘checklist’ risk screening (i.e. current standard of care).

- Health care utilisation objective:
  4. To summarise characteristics of participants’ interaction with the health system (e.g. antenatal visits, referrals to higher level of care), as well as use of aspirin and calcium, in women who underwent pre-eclampsia risk screening.

- Prevalence objective:
  5. To estimate prevalence of selected trial outcomes, in order to inform randomized trial design.

## Methods

### Overview of study design

**Fig. 1:**
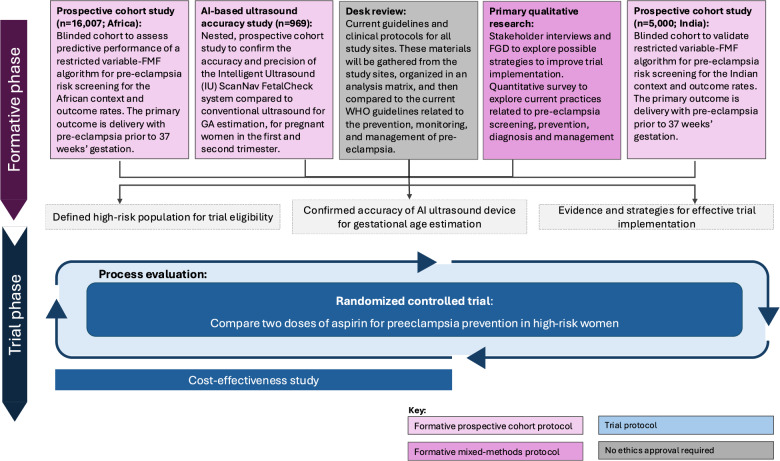
Overview of the PEARLS research activities. The prospective cohort study for predictive performance of risk screening is outlined in this protocol

The prospective cohort study described in this protocol is one of several formative research activities we are conducting prior to the PEARLS trial (Fig. 1). The additional activities (protocols reported elsewhere) include mixed-methods research with providers, women and health systems stakeholders, [37] and a nested sub-study on accuracy of a novel point-of-care ultrasound device for gestational age estimation. In addition, a prospective cohort in India will be added using the same methodology as this protocol, before the randomized trial is initiated there. Collectively these activities will inform and optimise the PEARLS Trial, which includes a process evaluation and cost-effectiveness analysis.

This multi-country, prospective cohort study will be conducted and reported in accordance with the TRIPOD 2024 standards [38]. The study aims to recruit 16,007 pregnant women at participating health facilities across Kenya, Ghana and South Africa. Eligible women < 20 weeks’ gestation will be approached, screened, consented and recruited at these facilities. At this initial visit, trained research midwives will collect history, medical and obstetric data pertaining to pre-eclampsia risk prediction, as well as obtaining an accurate blood pressure measurement using a validated device. Women will receive usual care during pregnancy and will be followed until birth.

### Study setting

This study will take place in Ghana, Kenya and South Africa. Principal Investigators in Ghana are based at the University of Ghana Medical School. The fieldwork will take place in two tertiary referral hospitals, ten district hospitals and four primary health care facilities in the Greater Accra Region. The Kenyan team are based at the University of Nairobi in Nairobi. The field work will take place in five tertiary/county level hospitals and 15 sub-county hospitals across several districts including Meru Country, Kisii Country, Kiambu Country, Kakamega County and Kisumu City. Principal Investigators in Cape Town are based at the University of Cape Town. The fieldwork will take place in the City of Cape Town at one tertiary referral hospital, two regional hospitals, one district hospital, and seven midwife obstetric units; each with several satellite basic antenatal care (BANC) clinics. The full list of facilities can be found in Appendix A.

In Ghana, hypertensive disorders are the second leading cause of maternal mortality. It is thought that pre-eclampsia affects 6.5% – 7.0% of pregnant women in Ghana, making it one of the top five leading causes of maternal and neonatal deaths in the country [39]. In 2017, the Kenyan Ministry of Heath produced their first Saving Mothers’ Lives report, an enquiry into maternal deaths in county and national referral hospitals in Kenya during 2014. Hypertensive disorders of pregnancy (including pre-eclampsia) was the third-leading cause of maternal mortality, accounting for 20% of maternal deaths nationwide [40]. The prevalence of pre-eclampsia in Kenya is estimated to be 5.6—6.5%, although this may under-represent women in rural areas. The most recent South African ‘Saving Mothers’ triennial report, by the National Committee for Confidential Enquiry into Maternal Deaths (NCCEMD), identified hypertensive disorders of pregnancy as causing 17.1% of all maternal deaths in 2022, making it the third most common cause [41]. In South Africa, pre-eclampsia affects an estimated 5% of pregnant women, and about 12% of women who are pregnant for the first time [42].

### Participants

The population eligible for the study are pregnant women with a viable pregnancy, presenting for an antenatal visit from 11 weeks and 0 days to < 20 weeks’ gestation at participating facilities, who are willing, able and consent to a follow-up cohort study. To be eligible, the woman must intend to deliver at a facility participating in PEARLS.

### Participant enrolment and timeline

Pregnant women attending participating facilities for antenatal care will be pre-screened, to identify those who are thought to be less than 24 weeks (i.e. 6 months) gestation – these are potentially eligible women.

All potentially eligible women will be invited to provide informed consent and undergo formal eligibility screening. As part of this screening, a point-of-care ultrasound for gestational age assessment will be used. This is to ensure that the gestational age estimate is based on an ultrasound as a study procedure. Eligible, consenting women will then be enrolled.

At time of enrolment, we will collect data on pre-eclampsia risk factors, which includes medical and obstetric history, mean arterial pressure, BMI and gestational age. These data reflect the variables used by the Tommy’s tool, which uses the FMF algorithm for pre-eclampsia risk assessment (see Table 3) [30]. Clinical management of enrolled women (including women with risk factors) will be in accordance with national guidelines. Pre-eclampsia risk scores will not be communicated to women or providers during this study.

**Table 3:** Variables collected within the Tommy’s Tool for pre-eclampsia risk screening [30]:

• Maternal factors—height, weight, BMI, racial origin, smoked or used tobacco in last 12 months, infertility treatment, family history of pre-eclampsia
• Medical history—high blood pressure, type I or type II diabetes, autoimmune disorders
• Obstetric history—parity, previous pre-eclampsia, stillbirth or fetal growth restriction in previous pregnancy
• Gestational age at time of screening, based on an ultrasound
• Blood pressure measurement using a calibrated, validated device (CRADLE device)

Participants will be followed until the pregnancy has concluded, and the occurrence of pre-eclampsia (or not) is ascertained. Enrolled women will be invited for an in-person, follow-up visit around 36 weeks’ gestation, and data will also be collected around time of birth. At the 36-week visit and the birth visit, research staff will measure blood pressure using the CRADLE device and perform a dipstick test for urine protein. For any participants admitted to hospital during their pregnancy, data will be collected on the reason and outcomes of this admission. Any suspicion or diagnosis of pre-eclampsia will not be concealed from women or clinicians. As part of outcome verification, we will identify enrolled women in whom a pre-eclampsia diagnosis is possible or suspected – these cases undergo independent medical review. Women who have not developed pre-eclampsia by the final study visit (i.e. birth visit) will form the group with ‘no pre-eclampsia’ outcome.

### Study endpoints

The endpoint for the primary objective (calibration) is the pre-eclampsia risk threshold calculated by the FMF algorithm (e.g. 1 in 30) that equates to a screen-positive rate of 10%. The endpoints for the secondary prognostic objectives are delivery with pre-eclampsia < 37 weeks’ gestation, as well as < 32, < 34, ≥ 37 weeks’ gestation.

For the secondary health care utilisation objective, endpoints are number of antenatal visits, number of referrals to higher-level care, as well as self-reported use of aspirin and calcium supplementation. For the secondary prevalence objective, we will collect data on several maternal and newborn endpoints, including maternal and perinatal mortality, interventions to manage PPH and other severe maternal morbidity, preterm birth, and process-of-care measures such as use of aspirin, calcium and anti-hypertensives, and referrals to higher level care (See Appendix B for operational definitions of these outcomes).

### Sample size requirements

Sample size calculations were based on the secondary objective for prognostic performance. The prevalence of delivery with preterm pre-eclampsia in the ASPRE Trial was 0.7% [43]. In a pooled analysis of 61,174 women screened with the FMF algorithm, the prevalence of preterm pre-eclampsia was 0.8% in all women and 1.8% (183/10,164) in Afro-Caribbean women [22]. For this study, we assumed a prevalence of 2% in the screened population. In pooled FMF studies in European countries, for Afro-Caribbean women a screen-positive rate of 10% equated to a risk cut-off of 1 in 20 and a sensitivity of 73.2% (66.4% to 79.1%) for preterm pre-eclampsia [22]. We have thus used 75% sensitivity to guide sample size calculations.

Under these assumptions, the required sample size for a precision of ± 5% is 14,406 women. We anticipate 288 women with the primary outcome will be detected. Factoring an additional 10% for any loss to follow up, 16,007 women are required. Thus, each country will aim to enroll 5,336 women. In the event the prevalence and/or detection rate is lower than anticipated, precision will be wider than ± 5%.

### Data collection and management

All data are collected by trained research midwives into a standardised digital form within a good clinical practice (GCP)-compliant, web-based data management platform (REDCap). Study data will be directly entered into REDCap using a device with reliable internet access. Offline data entry is available in REDCap using the REDCap Mobile App on phone/tablet devices, for sites where internet is not reliably available. During screening, we will also collect ultrasound scan information using an experimental, point-of-care ultrasound device. Study data will be managed centrally by the data management team at Burnet Institute using REDCap hosted on Burnet Institute servers. To calculate risk scores, anonymized risk screening data will be shared with the team managing the Tommy’s Tool, based in the UK. Each study site is responsible for data entry, as well as investigating and responding to data queries. The data management team will monitor range, consistency, and periodic reviews of distributions and identification of outliers. Regular feedback to sites will be used for quality assurance and to improve processes.

### Data analysis

The statistical analysis plan for this study is provided in Appendix C. Baseline characteristics of participants will be described using means and standard deviations for normally distributed variables, medians and 25th and 75th percentiles for non-normally distributed variables, and frequencies and percentages for binary and categorical variables.

Analysis for the primary calibration endpoint will estimate the screen positive rate as a binomial proportion (with 95% confidence intervals) over suitable risk threshold intervals provided by the restricted-variable FMF algorithm (e.g. 0.1 percentage points, as needed). The risk threshold that equates to a mean screen positive rate of 10% (i.e. the 10th percentile of the predicted risk values) will be determined. Measures of prognostic performance at this risk threshold will be determined as binomial proportions (with 95% confidence intervals). In addition to the 10% risk threshold, the detection rate (i.e. sensitivity), false positive rate and screen-positive rate for different cutoffs (e.g. 1 in 30, 1 in 50, etc.) will be determined. The rationale for our interest in 10% as risk threshold is based on previous studies of FMF algorithm-based risk screening in high-income countries which have produced screen-positive rates of 10–15% [18, 22]. Additionally, the ASPRE trial demonstrated efficacy of 150 mg aspirin vs placebo in high-risk women, which used FMF algorithm-based risk screening and had a screen-positive rate of 11.0% [43]. However, we will explore prognostic performance at other risk thresholds, to inform the decision on how best to define high-risk women for the trial phase.

For the secondary prognostic objective, we will use binomial proportions (with 95% confidence intervals) to estimate the measures of prognostic accuracy of the restricted-variable FMF algorithm for preterm pre-eclampsia—i.e. the detection rate (sensitivity), false positive rate, positive and negative predictive values (PPV, NPV) of the test at the risk threshold determined in the primary objective. To determine the overall predictive performance (discrimination and calibration) of the restricted-variable FMF algorithm for preterm pre-eclampsia, we will calculate the C-statistic (where a value of 1 indicates perfect discrimination and 0.5 indicating no discrimination beyond chance), calibration (where 1 is ideal calibration and a slope < 1 indicates overfitting), and calibration-in-the-large (ideal value of 0). Calibration plots will be presented, where the women will be grouped based on deciles of the predicted risk. Decision curves will also be presented that show the net benefit (i.e. benefit versus harm, calculated from the number of women, number of true positives, number of false positives, and the threshold probability) over a range of clinically relevant risk thresholds. Analyses for the remaining secondary prognostic objective will proceed similarly, using the secondary endpoints of delivery with pre-eclampsia < 32, < 34 and ≥ 37 weeks’ gestation.

Secondary objective 3 analyses will proceed as for the prognostic secondary objectives 1 and 2, with the results stratified by subgroups defined by women who undergo the index test at gestational ages (11–13 weeks; 14–16 weeks; 17- < 20 weeks). We will also descriptively compare prognostic results to history-based risk factor screening approaches in current national guidelines, using receiver operating characteristic (ROC) curves, the area under the ROC curves (AUCs) (C-statistic), over suitable risk threshold intervals (e.g. 0.1 percentage points, as needed). Calibration plots will also be compared.

For secondary objective 4, we will estimate the relevant population-level binomial proportions (for binary data, e.g. referral to high-level care, aspirin use, calcium use), medians (for count data, e.g. number of antenatal visits) with the method used to depend on the distribution of the count data, with 95% confidence intervals.

We pre-plan interim analyses for these objectives at regular intervals (e.g. when each country reaches 50% of their sample size). When the final sample size is reached, the database will be locked, and final analyses conducted.

#### Missing data

We anticipate that the proportion of missing data is likely to be low (e.g. 10% at a maximum), and loss to follow-up is accounted for in the sample size calculation. However, prospective cohorts with pregnant women can be affected by women attending non-study facilities for antenatal or birth care or relocating during pregnancy. To reduce missing data, we will contact women via phone before their 36 week follow-up, track women as they are referred to higher levels of care, and engage where possible with non-PEARLS facilities (particularly for any participants delivering in those facilities). Complete case analysis will be used for all objectives. In the unlikely event of a high proportion of missing outcome data (e.g. over 10%) we will conduct a sensitivity analysis to evaluate whether there are meaningful patterns of missingness to determine the best strategy for proceeding with the final analysis.

### Ethical considerations

Research staff at study sites will be trained on informed consent processes. An information sheet will be provided to participants. The language used in this sheet is non-technical and easy to understand. Participants will be given sufficient time to reflect on the information and will be given an opportunity to ask any questions.

If willing to participate, the participant will sign the consent form. This consent will be counter-signed by the research staff. Participants will be free to withdraw from the study at any stage without loss of benefits, and women will have the choice of including their data in analysis to the point of withdrawal, or exclusion of any data from analysis. If a woman cannot read or write, an impartial witness will be present during the entire informed consent reading and discussion. The impartial witness will also sign and date the consent form, along with the individual who performed the informed consent discussion. Participants will be provided with a study contact telephone number if they require further information or assistance. There will be no payment for participation, however some sites may reimburse participants for their travel costs.

## Discussion

The PEARLS formative research aims to inform the design of the PEARLS randomised trial, which will identify the most effective and safe dose of low-dose aspirin for pre-eclampsia prevention. To implement this trial, identification of women at high risk of preterm pre-eclampsia—and thus those women that would benefit from low-dose aspirin—is critical. The prospective cohort described in this protocol will determine the prognostic accuracy of a restricted-variable, FMF-based risk screening algorithm. This will be the first cohort to validate FMF-based risk screening in sub-Saharan Africa. By implementing and validating systematic risk screening in our settings, this formative prospective cohort will not only inform the design of the PEARLS trial but will provide vital evidence for implementation of systematic risk screening for pre-eclampsia in African women.

We expect that this prospective cohort will demonstrate that a modified version of the FMF algorithm, with maternal factors and MAP only, can identify women at high risk of preterm pre-eclampsia with reasonable accuracy. FMF has not been validated nor implemented at scale in any African country. Prior evidence suggests that FMF risk screening may have a different prognostic performance in women of Afro-Caribbean background living in high-income European countries, compared to Caucasian Europeans [22]. This study will provide critical evidence for implementation of risk screening in the PEARLS trial, including screen positive rates, sensitivity and specificity of modified FMF based screening in African settings, false positive rates, and appropriate risk thresholds. In addition, it will provide baseline prevalence of key trial outcomes such as preterm pre-eclampsia, PPH and PPH management interventions, to guide trial sample size decisions.

This cohort study will also collect data on healthcare utilisation, such as baseline use of interventions to prevent (aspirin, calcium) and treat pre-eclampsia (anti-hypertensives, magnesium sulfate, timing of birth), how pre-eclampsia is diagnosed, and management of high-risk women including referrals to higher care and number of antenatal visits. These baseline data will ensure efficient trial design and implementation. The logistics of trial implementation, such as site selection, allocation of resources and staff will be driven by the findings of the cohort. The formative research, including both this cohort and the additional activities described in Fig. 1, will inform any modifications to our digital risk screening tool (an adapted version of the Tommy’s Tool). Thus, the current study is critical to trial recruitment, and also to future implementation activities of systematic risk screening for pre-eclampsia in African countries.

Challenges in conducting prospective longitudinal studies on pre-eclampsia risk-screening and prevention have been discussed previously [44]. These include the lack of consistency in the diagnosis of pre-eclampsia, and quality of medical notes recording diagnostic criteria. The cohort studies used to develop the FMF algorithm relied on retrospective review of medical notes [22]. However, diagnosis of pre-eclampsia can be challenging in resource limited settings [44, 45]. To address these challenges, we will implement a stringent pre-eclampsia diagnostic review process, which involves expert obstetricians reviewing every case of possible pre-eclampsia, including both the data collected by the PEARLS staff during the duration of the study and the medical notes from each PEARLS facility at the time of delivery. This ensures we will generate the most accurate data on rates of pre-eclampsia in our settings.

This prospective cohort will generate the first evidence on the accuracy and use of an FMF-based risk screening algorithm in African settings. This will not only provide evidence that is critical to the implementation of risk screening in the PEARLS trial but will also provide the platform through which an FMF-based digital risk screening tool can be implemented at scale in Ghana, Kenya, South Africa and other African countries. These findings will be disseminated to health care providers, Ministries of Health, guideline developers and international health organisations to inform pre-eclampsia risk screening and prevention in LMICs.

Pre-eclampsia remains a leading cause of maternal mortality globally, and 99% of these deaths occur in LMICs [1, 3]. Interventions to prevent pre-eclampsia, such as low-dose aspirin, are poorly implemented in many resource-limited settings, in large part due to failures to identify women at high risk [44, 46]. By validating an effective risk screening algorithm, and tailoring this process to African contexts, we can make substantial gains in the prevention of pre-eclampsia and associated maternal and newborn morbidity and mortality.

## Supplementary Information

Supplementary Material 1.

Supplementary Material 2.

Supplementary Material 3.

## Data Availability

No datasets were generated or analysed during the current study.

## Abbreviations

ACOG: American college of obstetrics and gynecology
ASPRE: Combined multimarker screening and randomized patient treatment with aspirin for evidence: based pre-eclampsia prevention
BANC: Basic antenatal care
BMI: Body mass index
FMF: Fetal medicine foundation
GCP: Good clinical practice
HIV: Human immunodeficiency virus
ISSHP: International society for the study of hypertension in pregnancy
LMIC: Low- and middle-income country
MAP: Mean arterial pressure
NCCEMD: National committee for confidential enquiry into maternal deaths
NICE: National institute for health and care excellence
NPV: Negative predictive value
PAPP-A: Pregnancy associated plasma protein-A
PEARLS: Preventing pre-eclampsia: evaluating aspirin low-dose regimens following risk screening
PLGF: Placental growth factor
PPH: Postpartum haemorrhage
PPV: Postive predictive value
ROC: Receiver operating characteristic
TB: Tuberculosis
UTPI: Uterine artery Doppler pulsatility index
WHO: World health organisation
