# Supplementary material for: Protocol for a prospective cohort study on pre-eclampsia risk prediction in Ghana, Kenya and South Africa
Source: Reprod Health. 2025 Oct 27;22:209. doi: 10.1186/s12978-025-02156-1 (PMC12560518; doi:10.1186/s12978-025-02156-1)
Supplement: Supplementary file 1 — Supplementary Material 1. [file 12978_2025_2156_MOESM1_ESM.docx]

## Appendix A - List of study sites

| Ghana | |
| --- | --- |
| Tertiary referral hospitals | Korle Bu Teaching Hosp |
|  | Greater Accra Regional Hospital |
| District Hospitals | Achimota Hospital |
|  | Mamprobi Hospital |
|  | Maamobi General Hospital |
|  | LEKMA Hospital |
|  | Ga West Municipal Hospital (Amasaman) |
|  | Ga South Municipal Hospital (Weija) |
|  | Shai Osudoku Hospital |
|  | Tema General Hospital |
|  | Ashaiman Municipal Hospital |
|  | Ada East District Hospital |
| Primary Health Facility | Usher Maternity Home (James Town) |
|  | Kaneshie Polyclinic |
|  | Osu Government Maternity Home |
|  | Tema Polyclinic |
| Kenya | |
| Tertiary referral hospitals | Muranga |
|  | Meru Teaching and Referral Hospital |
|  | Kiambu |
|  | Kakamega Teaching and Referral Hospital |
|  | Jaramogi Oginga Odinga Teaching and Referral Hospital |
| Sub-country hospitals | Ruiru |
|  | Gatundu |
|  | Igegania |
|  | St Mary’s Mumias Hospital |
|  | Butere |
|  | Malava |
|  | Kisumu County hospital |
|  | Lumumba |
|  | Ahero |
|  | Maragua, |
|  | Kagumo |
|  | Kangema |
|  | Nyambene |
|  | Kiirua |
|  | Nkubu Mission Hospital |
| South Africa | |
| Hospitals | Mowbray Maternity |
|  | New Somerset |
|  | Grootshuur |
|  | Mitchells Plain |
| Midwife Obstetric Unit (MOU) | Guguleta |
|  | Mitchells Plain |
|  | Hanover Park |
|  | Retreat |
|  | False Bay |
|  | Vanguard |
|  | Dunoon |
| Basic Antenatal Care Clinics | Tafelsig (MPMOU) |
|  | Westridge (MPMOU) |
|  | Vuyani (GMOU) |
|  | Masicendane (GMOU) |
|  | Heideveld (HvPMOU) |
|  | Silvertown (HvPMOU) |
|  | Hout Bay (RMOU) |
|  | Grassy Park (RMOU) |
|  | Masiphumulele (FBH) |
|  | Fishoek (FBH) |
|  | Langa (VH) |
|  | Bonteheuvel (VH) |
|  | Maitland (DN) |
|  | Factreton (DN) |
|  | Lentegeur (MPDH) |
|  | District Six (NSH) |
|  | Spencer road (NSH) |
|  | Liesbeek (MMH) |
