# Supplementary material for: Protocol for a prospective cohort study on pre-eclampsia risk prediction in Ghana, Kenya and South Africa
Source: Reprod Health. 2025 Oct 27;22:209. doi: 10.1186/s12978-025-02156-1 (PMC12560518; doi:10.1186/s12978-025-02156-1)
Supplement: Supplementary file 2 — Supplementary Material 2. [file 12978_2025_2156_MOESM2_ESM.docx]

**Appendix B**

**Table 1. Clinical outcome definitions for Objective 1B: Prognostic accuracy study**

| **Primary outcomes:** | **Operational definition and measurement** |
| --- | --- |
| ***Clinical - Maternal*** | |
| Delivery with preeclampsia prior to 37 weeks’ gestation | Delivery with preeclampsia before 37 weeks of gestation. Preeclampsia is defined according to ICD-11 and ACOG guidelines (see below).  In instances of clinical uncertainty regarding presence of preterm preeclampsia, these cases will be reviewed by a study obstetrician to confirm or exclude preeclampsia diagnosis. |
| Preeclampsia | Pre-eclampsia is defined using the ICD-11 definition of pre-eclampsia.  Specific operational definitions are derived from the ACOG definition for pre-eclampsia.  In ICD-11, pre-eclampsia is defined as: new-onset hypertension with proteinuria, OR in the absence of proteinuria, evidence of new-onset maternal end-organ dysfunction OR neurological conditions OR fetal growth restriction.  Among women with chronic (pre-existing) hypertension at time of recruitment, it is defined as development of new proteinuria, OR in the absence of proteinuria, evidence of new-onset maternal end-organ dysfunction OR neurological conditions OR fetal growth restriction.  In this study, testing for presence of maternal end-organ dysfunction (such as platelet count and liver function) is based on clinician judgement and test availability. Routine screening for end-organ dysfunction is not expected.  ***Operational definition (i.e. specific endpoint definition):***  *Blood pressure:*   - *New-onset hypertension:* Systolic blood pressure of 140 mm Hg or more or diastolic blood pressure of 90 mm Hg or more after 20 weeks of gestation in a woman with a previously normal blood pressure.​ - *New-onset severe hypertension:* Systolic blood pressure of 160 mm Hg or more or diastolic blood pressure of 110 mm Hg or more. (Severe hypertension can be confirmed within a short interval (minutes) to facilitate timely antihypertensive therapy).   *Proteinuria:  ​*   - 300 mg or more per 24-hour urine collection (or this amount extrapolated from a timed collection) OR​ - Protein/creatinine ratio of 0.3 or more OR - Dipstick reading of 2+ (used only if other quantitative methods not available) ​   ​  *Maternal end-organ dysfunction:*  Testing for presence of maternal end-organ dysfunction (such as platelet count and liver function) is based on clinician judgement and test availability. Routine screening of all participants for end-organ dysfunction is not expected.  This can include:   - Thrombocytopenia (platelet count less than 100 x 10^9^) - Renal insufficiency: Serum creatinine concentrations greater than 1.1 mg/dL - Impaired liver function: Elevated blood concentrations of liver transaminases to twice normal concentration - Pulmonary edema   *Neurological conditions:*   - New-onset headache unresponsive to medication and not accounted for by alternative diagnoses, or visual disturbances.   *Fetal growth restriction:*   - Birthweight less than the 3^rd^ percentile |
|  |  |
| Delivery with preeclampsia prior to 32 weeks’ gestation | Delivery with diagnosed preeclampsia before 32 weeks of gestation. |
| Delivery with preeclampsia prior to 34 weeks’ gestation | Delivery with diagnosed preeclampsia before 34 weeks of gestation. |
| Delivery with preeclampsia at or beyond 37 weeks’ gestation | Birth of a neonate at or beyond 37 weeks’ gestation in a woman diagnosed with preeclampsia. |
| Maternal death | Any maternal death in a participant, from time of enrolment to end of study |
| Eclampsia | Convulsions, seizures or coma in pregnant women associated with hypertension, edema and/or proteinuria |
| Placental abruption | Detachment of the placenta from the uterine wall. Clinical signs can include:   - Vaginal bleeding - Uterine tenderness - Tetanic uterus - Fetal distress - Maternal tachycardia - Hypofibrogenemia     Assessment is made by clinical examination of the woman by an obstetric physician. |
| Severe maternal morbidity (composite outcome) | Any one or more of the following, measured based on clinical diagnosis:   - Eclampsia - Cerebrovascular accident (stroke); - Visual disturbances - Pulmonary oedema; - Acute kidney injury; - Liver capsule hematoma or rupture; - Renal failure; - liver failure; - HELLP syndrome |
| Intensive care unit (ICU) admission | Reported as:   - Maternal admission to intensive care (any) - Maternal admission to intensive care for > 24 hours   Admission may be at any time between enrolment and end of study, measured as a binary yes/no outcome |
| Duration of ICU admission | Length of intensive care admission will be recorded in days, from day of admission until day of official discharge |
| Intubation and mechanical ventilation | Intubation for the purpose of mechanical ventilation of the mother for reasons other than childbirth, measured as a binary yes/no outcome |
| Severe hypertension | Systolic blood pressure of 160 mm Hg or more and/or diastolic blood pressure of 110 mm Hg or more. |
| Composite outcome on use of additional interventions for management of primary PPH (regardless of mode of birth) | Composite outcome will include any women that required any of the following up until end of study:   1. Use of additional uterotonics for PPH treatment OR 2. Use of tranexamic acid for PPH treatment OR 3. Use of invasive non-surgical interventions for PPH treatment (including uterine tamponade [balloon or suction] or non-pneumatic antishock garment use) OR 4. Use of surgical interventions for PPH treatment (including laparotomy, B-lynch suture, uterine artery ligation, or hysterectomy) OR 5. Use of blood transfusion |
| ***Clinical - Fetal/newborn*** | |
| Pregnancy loss prior to 22 weeks’ gestation | Any pregnancy loss in an enrolled woman, due to either spontaneous loss or termination of pregnancy |
| Stillbirth | Any fetal death (stillbirth) in an enrolled woman, occurring from 22 weeks or later. |
| Very early neonatal death | Death of the baby within 24 hours of birth |
| Perinatal death | Any stillbirth or very early neonatal death |
| Admission to NICU/SNCU at 24h | Newborn admitted in the neonatal intensive care unit (NICU) or Special care unit (SNCU) at 24 hours after birth |
| Very early neonatal death or admission to NICU/SNCU at 24 h (composite) | Composite outcome of death of the baby within 24 h of birth OR baby admitted in the NICU/SNCU at 24 h after the birth. The denominator for this outcome is all live born babies. |
| Gestational age at birth | Calculated based on the gestational age at time of enrolment, date of enrolment and date delivery |
| Early preterm birth (<34 weeks’) | Delivery before 34 weeks of gestation based on dating ultrasound in any enrolled woman |
| Preterm birth (<37 weeks’) | Delivery before 37 weeks of gestation based on dating ultrasound in any enrolled woman |
| Birthweight | Measured in first 24 hours of life whilst naked, in grams using a standardized, calibrated scale.  All babies regardless of vital status will be weighed after birth. |
| Low birthweight | Birthweight less than 2500g, measured in first 24 hours of life whilst naked, in grams using a standardized, calibrated scale. |
| Small-for-gestational age | Birthweight less than the 10^th^ percentile for gestational age, based on INTERGROWTH-21 charts. |
| **Healthcare utilisation** | |
| Number of antenatal visits | Number of times a women visited antenatal care between recruitment and birth |
| Referral to higher-level care | Woman referred to a higher level of care, measured as a binary yes/no outcome.  This will be reported separately as:   - Referral to higher level of care during pregnancy - Referral to higher level of care at delivery |
| Self-reported use of aspirin | Woman used aspirin during her pregnancy, measured as a binary yes/no outcome  If yes, dose and frequency of use of aspirin |
| Self-reported use of calcium supplementation | Woman used calcium during her pregnancy, measured as a binary yes/no outcome  If yes, dose and frequency of use of calcium |
