# Supplementary material for: Protocol for a prospective cohort study on pre-eclampsia risk prediction in Ghana, Kenya and South Africa
Source: Reprod Health. 2025 Oct 27;22:209. doi: 10.1186/s12978-025-02156-1 (PMC12560518; doi:10.1186/s12978-025-02156-1)
Supplement: Supplementary file 3 — Supplementary Material 3. [file 12978_2025_2156_MOESM3_ESM.docx]

Statistical Analysis Plan

| STUDY FULL TITLE | Formative research for the PEARLS Trial (Preventing pre-eclampsia: Evaluating AspiRin Low-dose regimens following risk Screening) |
| --- | --- |
| PROTOCOL VERSION | Version 3.0  Dated 5^th^ June 2024 |
| CHIEF INVESTIGATORS | Professor Joshua Vogel (Implementation Lead), Burnet Institute  Professor Metin Gülmezoglu (Sponsor), Concept Foundation |
| STATISTICIANS | Professor Julie Simpson, University of Melbourne  Dr Rob Mahar, University of Melbourne  Dr Digsu Koye, University of Melbourne |
| SAP AUTHORS | Professor Julie Simpson, University of Melbourne  Dr Rob Mahar, University of Melbourne  Dr Digsu Koye, University of Melbourne |
| SAP VERSION | 1.0 |
| SAP VERSION DATE | 19 December 2024 |

**SAP Revision History**

| **Version** | **Reason(s) for change** | **Date** |
| --- | --- | --- |
| 1.0 | Initial version | 19 December 2024 |
|  |  |  |
|  |  |  |
|  |  |  |
|  |  |  |

**SAP Signatures**

I give my approval for the attached SAP entitled Vogel_Josh_PEARLS_SAP_v1.0 dated 19 December 2024.

| **Name / Affiliation** | **Signature** | **Date** |
| --- | --- | --- |
| Professor Joshua Vogel, Burnet Institute  (Implementation Lead) |  |  |
| Professor Metin Gülmezoglu, Concept Foundation (Sponsor) |  |  |

**SAP Authors**

| **Name / Affiliation** | **Signature** | **Date** |
| --- | --- | --- |
| Prof Julie Simpson  (Senior statistician) |  |  |
| Dr Rob Mahar  (Senior statistician) |  |  |
| Dr Digsu Koye  (Statistician) |  |  |

Table of Contents

[Abbreviations and Definitions 5](#_Toc202008298)

[1 Introduction 6](#_Toc202008299)

[2 Analysis Objectives and Endpoints 7](#_Toc202008300)

[2.1 Analysis Objectives 7](#_Toc202008301)

[2.1.1 Objective 1B - Prognostic accuracy and predictive performance of pre-eclampsia risk screening 7](#_Toc202008302)

[2.2 Endpoints 8](#_Toc202008303)

[2.2.1 Objective 1B - Prognostic accuracy and predictive performance of pre-eclampsia risk screening 8](#_Toc202008304)

[3 Study Methods 10](#_Toc202008305)

[3.1 Study Design 10](#_Toc202008306)

[3.1.1 Objective 1B - Prognostic accuracy and predictive performance of pre-eclampsia risk screening 10](#_Toc202008307)

[4 Sample size 11](#_Toc202008308)

[5 Analysis sets/Subgroups 12](#_Toc202008309)

[5.1 Objective 1B - Prognostic accuracy and predictive performance of pre-eclampsia risk screening 12](#_Toc202008310)

[6 Handling of Missing Values and Other Data Conventions 13](#_Toc202008311)

[7 Statistical Methodology 13](#_Toc202008312)

[7.1 Objective 1B - Prognostic accuracy and predictive performance pre-eclampsia risk screening 13](#_Toc202008313)

[8 Sensitivity Analyses 17](#_Toc202008314)

[9 Interim Analysis 17](#_Toc202008315)

[10 QC Plans 18](#_Toc202008316)

[11 Programming Plans and allocation of who will complete the statistical analyses 18](#_Toc202008317)

[12 References 18](#_Toc202008318)

[13 Appendix 19](#_Toc202008319)

# Abbreviations and Definitions

| **Abbreviation** | **Definition** |
| --- | --- |
| AUC | Area under the receiver operating characteristic curve |
| DSMB | Data Safety Monitoring Boards |
| FMF | Fetal Medicine Foundation |
| GA | Gestational age |
| ICU | Intensive Care Unit |
| MISCH | Methods and Implementation Support for Clinical and Health research Hub |
| NPV | Negative predictive value |
| PEARLS | Preventing pre-eclampsia: Evaluating AspiRin Low-dose regimens following risk Screening |
| PPH | Postpartum haemorrhage |
| PPV | Positive predictive value |
| PO | Primary objective |
| ROC | receiver operating characteristic curve |
| SAP | Statistical Analysis Plan |
| SO | Secondary objective |
| TRIPOD | Transparent Reporting of a Multivariable Prediction Model for Individual Prognosis or Diagnosis |
| WHO | World Health Organisation |

# 1 Introduction

Pre-eclampsia is a multi-system disorder that develops during pregnancy due to abnormal placentation, dysregulation of angiogenesis, inflammation, oxidative stress, and maternal systemic vascular dysfunction. It is diagnosed through identification of new-onset hypertension in the presence of either proteinuria or new-onset maternal organ dysfunction at or after 20 weeks’ gestation. Pre-eclampsia is a leading cause of maternal and neonatal morbidity and mortality, globally it accounts for an estimated 14% of the 287,000 maternal deaths that occur each year.

Prophylaxis during pregnancy with daily low-dose aspirin is currently the standard treatment for women identified as high risk for pre-eclampsia. However, there is uncertainty surrounding the safest and most effective dosage of aspirin in low-, middle-, and high-income countries. In WHO’s 2021 recommendations on aspirin for pregnant women, the need for a randomised trial comparing a 150 mg dose to 75mg (current standard of care) was a high research priority. There is currently uncertainty as to whether the higher dose has greater benefit (greater prevention of pre-eclampsia) or may increase the risk of maternal bleeding.

This formative research project precedes, and will inform, the PEARLS (Preventing pre-eclampsia: Evaluating AspiRin Low-dose regimens following risk Screening) trial in Ghana, Kenya and South Africa. This formative phase aims to optimise pre-eclampsia risk screening, gestational age (GA) estimation, trial participation and clinical care pathways, evaluate baseline prevalence of primary trial outcomes, and identify potential barriers to trial implementation.

This statistical analysis plan (SAP) contains details of the planned statistical analyses to be completed to support the formative prospective cohort of the PEARLS study.

# 2 Analysis Objectives and Endpoints

## 2.1 Analysis Objectives

The formative phase of the PEARLS study aims to optimise pre-eclampsia risk screening and clinical care pathways, evaluate baseline prevalence of primary trial outcomes, and identify potential barriers to trial implementation. These findings will inform conduct of the main trial. The aims of this study are outlined below.

### 2.1.1 Objective 1B - Prognostic accuracy and predictive performance of pre-eclampsia risk screening

This study aims to evaluate the prognostic accuracy and predictive performance of pre-eclampsia risk screening using the restricted-variable Fetal Medicine Foundation (FMF) screening, embedded within an adapted version of Tommy’s Tool.

The restricted-variable FMF screening test includes the following variables: weight, height, racial origin, smoked cigarettes in the last 12 months, infertility treatment in this pregnancy, mother or sister with pre-eclampsia, high blood pressure outside pregnancy, type I or II diabetes, autoimmune disorders (Lupus, Antiphospholipid syndrome, Rheumatoid conditions, Hashimotos, Crohns, etc), first pregnancy (yes/no), previous preeclampsia, previous baby weighed less than 2kg, history of stillbirth/ pregnancy loss ≥24 weeks and maternal blood pressure.

**Specific research objectives**

**Primary objective (PO)**

1B.PO1 To estimate a risk threshold that equates to a screen-positive rate of 10% (calibration objective).

**Secondary objectives (SO)**

Prevalence of trial outcomes

1B.SO1 Estimate prevalence of the PEARLS trial’s primary outcomes in participating hospitals.

Prognostic objectives

1B.SO2 To estimate the prognostic accuracy and predictive performance of the restricted-variable FMF screening test within an adapted version of Tommy’s tool, for the outcomes delivery with pre-eclampsia at <32 weeks, <34 weeks, <37 weeks (primary outcome of interest) and ≥ 37 weeks’ gestation.

1B.SO3 To estimate whether prognostic accuracy of the screening test varies at different gestational ages of testing (<14, <16 and <20 weeks).

1B.SO4 To summarise the prognostic accuracy of the restricted-variable screening test to history-based risk factor screening only using NICE/ACOG/other international agencies or current standard of care in Ghana, Kenya and South Africa. The risk factors used for the prediction of pre-eclampsia using different history-based risk factors, including the current standard of care in Ghana, Kenya, and South Africa, are provided in Table 1 in the Appendix.

Health care utilisation objective

1B.SO5 To summarise characteristics of participants interaction with the health system (e.g. antenatal visits, referrals to higher level of care), as well as use of aspirin and calcium, in women who underwent pre-eclampsia risk screening.

**Exploratory objective (EO)**

1B.EO1 To develop a new risk prediction model for delivery with pre-eclampsia before 37 weeks of gestation.

## 2.2 Endpoints

The endpoints for each of the objectives are listed below.

### 2.2.1 Objective 1B - Prognostic accuracy and predictive performance of pre-eclampsia risk screening

**Primary endpoint**

1B.PO1 The endpoint for the calibration objective is the pre-eclampsia risk threshold (e.g. 1 in 20, 1 in 30, etc) that equates to a screen positive rate of 10%.

**Secondary endpoints**

1B.SO1 The prevalence of the primary outcomes for the PEARLS trial will be estimated in the formative cohort. These outcomes are:

1. Benefit outcome: Delivery with pre-eclampsia before 37 weeks of gestation
2. Safety outcome: Composite outcome on use of additional, invasive interventions for management of primary PPH (regardless of mode of birth):
3. Use of additional uterotonics for PPH treatment OR
4. Use of tranexamic acid for PPH treatment OR
5. Use of invasive non-surgical interventions for PPH treatment (including uterine [balloon or suction] tamponade or non-pneumatic antishock garment use) OR
6. Use of surgical interventions for PPH treatment (including laparotomy, B-lynch suture, uterine artery ligation, or hysterectomy) OR
7. Use of blood transfusion

1B.SO2-1B.SO4

The endpoints for the prognostic objectives for this study are:

- Delivery with pre-eclampsia prior to 37 weeks’ gestation
- Delivery with pre-eclampsia prior to 32 weeks’ gestation
- Delivery with pre-eclampsia prior to 34 weeks’ gestation
- Delivery with pre-eclampsia at or beyond 37 weeks’ gestation.

1B.SO5 The endpoints for the health care utilisation objective are:

- Characteristics of participants interaction with the health system as measured by:
  - Number of antenatal visits
  - Gestational age at initial presentation to antenatal care
  - Referral to higher-level care
- Self-reported use of aspirin
- Self-reported use of calcium supplementation

In addition, we will collect data on several clinical endpoints for mother and fetus/baby. Operational definitions for these endpoints are provided in Table 2 in the Appendix. These data will be used to inform preparations for the randomised trial.

***Maternal secondary outcomes:***

1. Maternal death during enrolment/follow up period
2. Eclampsia
3. Placental abruption
4. Severe maternal morbidity - any one or more of: Eclampsia; cerebrovascular accident (stroke); visual disturbances; pulmonary oedema; acute kidney injury; liver capsule hematoma or rupture; renal failure; liver failure; HELLP syndrome.
5. Intensive care unit (ICU) admission: a) Any; b) for >24 hours; c) duration of ICU admission (hours).
6. Intubation and mechanical ventilation (not for childbirth)
7. Severe hypertension

***Fetal/newborn secondary outcomes:***

1. Pregnancy loss prior to 22 weeks’ gestation (either spontaneous loss or termination of pregnancy)
2. Stillbirth (fetal death from 22 weeks onward)
3. Very early neonatal death (status at 24 hours)
4. Perinatal death (any stillbirth or very early neonatal death)
5. Admission to NICU/SNCU at 24 h after birth
6. Very early neonatal death or admission to NICU/SNCU (composite)
7. Gestational age at birth (weeks)
8. Early preterm birth (<34 weeks’ gestation)
9. Preterm birth (<37 weeks’ gestation)
10. Birthweight (g), measured in the first 24 hours after birth using a calibrated scale
11. Low birthweight (<2500g)
12. Small for gestational age

# 3 Study Methods

## 3.1 Study Design

### 3.1.1 Objective 1B - Prognostic accuracy and predictive performance of pre-eclampsia risk screening

A prospective, blinded cohort study will be conducted to estimate the prognostic accuracy and predictive performance of the pre-eclampsia risk screening tool (i.e. index test). This study will follow the Transparent Reporting of a Multivariable Prediction Model for Individual Prognosis or Diagnosis (TRIPOD) reporting guideline for prognostic studies.^1^ This study will recruit 16,007 eligible, consenting women in Ghana, Kenya and South Africa (aiming for 5,336 women per country) who will undergo the pre-eclampsia risk screening process.

The population eligible for the study will be pregnant women presenting for an antenatal visit from 11 weeks and 0 days to <20 weeks’ gestation at participating facilities, who are willing, able and consent to a follow-up cohort study. To be eligible, the woman must intend to deliver at a participating facility and have no knowledge that the pregnancy is not viable (e.g., intrauterine fetal death or extrauterine pregnancy). Once recruited and assigned a unique study number, women undergo pre-eclampsia risk screening. These data are collected by a trained research midwife into a standardised digital form within Tommy’s Tool. Participants will be followed up during pregnancy until delivery occurs or pregnancy ends.

# 4 Sample size

**Objective 1B -** **Prognostic accuracy and predictive performance of pre-eclampsia risk screening**

The sample size calculations are based on the prognostic performance objective, specifically, the probability that an individual had a positive restricted-variable FMF screening test (index test) given they went on to preterm delivery (<37 weeks) with pre-eclampsia (i.e. primary outcome detection rate, also known as sensitivity).

The prevalence of the primary outcome (preterm delivery <37 weeks with pre-eclampsia) in the screened population from ASPRE Trial was 0.7%. In a pooled analysis of 61,174 women screened with FMF, the prevalence of preterm pre-eclampsia was 0.8% in all women and 1.8% (183 / 10164) in Afro-Caribbean women only.^2^ For this study, we assume that the prevalence of the primary outcome in the screened population at 2%.

In this study we will fix the screen-positivity rate at approximately 10%. Based on data from pooled European analyses, for Afro-Caribbean women this would equate to a risk cut-off of 1 in 20, and a primary outcome detection rate of 73.2% (66.4% to 79.1%).^2^ We have thus assumed a detection rate of the index test is 75%.

Under these assumptions, the required sample size for a precision of +/-5% is 14,406 women. We anticipate 288 women with the primary outcome will be detected. With 288 women with preterm pre-eclampsia, we have >10 events per predictor variable of the restricted variable FMF risk-screening test (total of 13 predictors). Factoring in an additional 10% for any loss to follow up, 16,007 women are required. This sample size is for the total number of pregnant women recruited from all three countries. Thus, each country will aim to screen and recruit 5,336 women; however total numbers per country may vary.

We also performed a precision-based sample size for the calibration objective, that is, estimation of a risk threshold that equates to a screen-positive rate of 10%. Weighting the multinomial distributions (combined over three studies) for risk scores for screened women with and without preterm pre-eclampsia found in Wright et al.^3^ by 14,406 and 288 (sample size numbers based on calculations above), respectively, and simulating 1,000 hypothetical studies, we found that the 10% screen positive rate (i.e. the upper 90^th^ percentile of the cumulative distribution of risk scores) will be estimated with 95% precision confidence limits of approximately +/- 0.5 percentage points. We expect similar precision for the PEARLS estimate of the risk threshold corresponding to the 90^th^ percentile, despite likely differences between the PEARLS and Wright et al. cohort.

An initial formal interim analysis will be conducted after approximately 67% of participants have been enrolled. This analysis will be for objective 1B, primary objective (1B.PO1), using only the screening/enrolment data to determine the threshold that gives a 10% positive screening rate. A precision-based sample size calculation (similar to the above calculation) suggests that the 10% screen positive rate will be estimated with 95% precision confidence limits of approximately +/- 0.6 percentage points.

# 5 Analysis sets/Subgroups

Different analysis sets will be used depending on the objectives, as described below.

## 5.1 Objective 1B - Prognostic accuracy and predictive performance of pre-eclampsia risk screening

The primary analysis of the endpoints for Objective 1B will include all pregnant women (singleton or multiple pregnancy) enrolled across the three African countries, with an acceptable ultrasound-based GA estimate. This can include either 1) GA estimated using an acceptable conventional ultrasound (prior, during or after enrolment), or 2) GA estimated using an AI-based intelligent ultrasound (IU) algorithm, provided that the IU device is demonstrably accurate. For analysis, each participant is assigned a single study GA, according to the most accurate ultrasound-based estimate available for that participant (i.e. ultrasounds earlier in pregnancy are generally more accurate).

If there is no acceptable ultrasound-based GA estimate, they are excluded from the analysis population on the basis that the primary outcome (preterm pre-eclampsia) cannot be reliably determined.

Women with pregnancy loss prior to 20 weeks’ gestation (either spontaneous loss or termination of pregnancy) will be excluded from the analysis.^4^ They will be reported in descriptive tables only.

Maternal or fetal deaths occurring from 20 weeks onwards will be accounted for using a competing risk approach. Maternal deaths, fetal deaths or preterm births clearly due/related to pre-eclampsia will be considered as having experienced a pre-eclampsia event, while delivery due to other causes will be regarded as competing events.

Predefined sub-group analysis will include:

- Women who undergo the index test at gestational ages (11-13 weeks; 14-16 weeks; 17- <20 weeks)
- Different modes of ultrasound used for estimating the study GA (conventional vs IU device)
- Hypertension at baseline

# 6 Handling of Missing Values and Other Data Conventions

**Objective 1B** - The proportion of missing data is likely to be low (e.g. 10% at a maximum), and loss to follow-up is accounted for in the sample size calculation. Complete case analysis will be used for all objectives. In the unlikely event of a high proportion of missing outcome data (e.g. over 10%) we will conduct a sensitivity analysis to evaluate whether there are meaningful patterns of missingness to determine the best strategy for proceeding with the final analysis.

Participants who were enrolled to the study but did not meet the eligibility criteria or enrolled without an informed consent/assent will be excluded from the analysis.

# 7 Statistical Methodology

Baseline characteristics of all study participants included in each of the studies will be described using means and standard deviations for normally distributed variables, medians and 25^th^ and 75^th^ percentiles for non-normally distributed variables, and frequencies and percentages for binary and categorical variables.

## 7.1 Objective 1B - Prognostic accuracy and predictive performance pre-eclampsia risk screening

**Primary objective**

Analysis for the primary calibration objective (1B.PO1)

Analysis for the primary calibration endpoint will estimate the screen positive rate as a binomial proportion (with 95% confidence intervals) over suitable risk threshold intervals provide by the restricted-variable FMF algorithm (e.g. 0.1 percentage points, as needed). The risk threshold that equates to a mean screen positive rate of around 10% (i.e. the 10^th^ percentile of the predicted risk values) will be provided, and measures of prognostic performance at this risk threshold will be provided as binomial proportions (with 95% confidence intervals). The risk threshold will be determined overall and by country.

The rationale for selection of 10% as risk threshold is based on:

1. Previous studies of FMF algorithm-based risk screening in high-income countries which have produced screen-positive rates of 10-15%.
2. The ASPRE trial which has demonstrated efficacy of 150mg aspirin vs placebo in high-risk women, which used FMF algorithm-based risk screening and had a screen-positive rate of 11.0%.
3. Consultations with PEARLS study team, regarding what screen-positive rate can be accommodated (or tolerated) by health systems in participating countries.

We will also describe and inspect the characteristics for the two groups of women – those identified as high-risk (10%) or not (90%). For example, the prevalence of chronic hypertension, diabetes, multiple pregnancy and other key characteristics in these two groups will be assessed.

In addition to the 10% risk threshold, detection rate, false positive rate and screen-positive rate for different cutoffs will be evaluated and results will be provided as a supplementary information.

**Secondary objectives**

Prevalence of trial outcomes

An estimate of the prevalence of primary outcomes (1B.SO1) in the high-risk population will be determined from all pregnant women (singleton or multiple pregnancy) enrolled across the three African countries. We will report the proportion of women who are screen-positive (i.e. high-risk women), and the relative difference in the prevalence of primary trial outcomes (benefit and safety) between those who screen positive and screen negative. These data will be used to confirm or revise sample size estimates for the main PEARLS trial.

Analysis for the prognostic secondary objective 2 (1B.SO2)

We will estimate using binomial proportions (with 95% confidence intervals) the measures of prognostic accuracy of the restricted-variable FMF algorithm for pre-eclampsia (<37 weeks), i.e. the detection rate, false positive rate, positive predictive value (PPV), and negative predictive value (NPV) of the test at the risk threshold determined in the primary objective. To determine the overall predictive performance (discrimination and calibration) of the restricted-variable FMF algorithm for predicting the primary endpoint (preterm pre-eclampsia), we will calculate the C-statistic (where a value of 1 indicates perfect discrimination and 0.5 indicating no discrimination beyond chance), calibration (where 1 is ideal calibration and a slope<1 indicates overfitting), and calibration-in-the-large (ideal value of 0).

Discrimination refers to the ability of the model to separate between women who develop preterm pre-eclampsia and those who do not.

Calibration refers to how well the predicted risk from the model agrees with the observed risks of preterm pre-eclampsia for women in the study. The calibration slope is the slope of the regression line fitted to the relationship (across all pregnant women) between predicted (x-axis) and observed (y-axis) risk probabilities. The ideal value of 1 indicates overall agreement between observed and predicted risks across the whole range of predicted values; a slope <1 indicates overfitting where predictions are too extreme compared to observed probabilities, and slope >1 indicates underfitting where predictions are too narrow).^5^

The calibration-in-the-large (ideal value of 0) summarises whether the model over-predicts or under-predicts risk probabilities on average across all women. It indicates whether risk predictions are systematically too high (calibration-in-the-large <0) or too low (calibration-in-the-large >0).

Calibration plots will be presented, where the women will be grouped based on deciles of the predicted risk of preterm pre-eclampsia and the observed risk calculated within each of these decile groupings. Internal validation will be performed using 10-fold cross-validation and bootstrap samples.

Decision curves will also be presented that show the net benefit (i.e. benefit versus harm, calculated from the number of women, number of true positives, number of false positives, and the threshold probability) over a range of clinically relevant threshold values of risk.^5^

Additional analyses similar to the prognostic secondary objective 1 above but using the secondary endpoints of delivery with pre-eclampsia <32, <34 and >= 37 weeks’ gestation will be conducted.

Analysis for secondary objective 3 (1B.SO3) will proceed as for the prognostic secondary objective 2 (1B.SO2), with the results stratified by subgroups defined by:

- Women who undergo the index test at gestational ages (11-13 weeks; 14-16 weeks; 17- <20 weeks)
- Different modes of ultrasound used for estimating the study GA (conventional vs IU device)

When the Tommy’s tool is used in clinical context in the UK, some subgroups of women receive a risk score that is below the cut-off for high-risk, but are classified as high-risk automatically for other reasons. This includes:

- Any woman with a history of stillbirth or fetal growth restriction
- Any woman with chronic hypertension or diabetes
- Any woman with a known multiple pregnancy

Additional analyses will be performed to ascertain the possible effects of including or excluding these ‘automatic high risk’ groups within the high-risk population. We will use the same prognostic endpoints and analyses as Objective 1B.SO2. These will inform the decision on how the trial population should be defined, and whether the use of ‘automatic high risk’ groups meaningfully affects detection, accuracy and primary outcome prevalence.

Additional analyses will be performed to explore the possible effects of method of ultrasound estimation for GA on prognostic performance. We will use prognostic endpoints for the scenarios of 1) all analysed women having their study GA defined by the IU device, and 2) all analysed women having their study GA defined by conventional F1 only.

Analysis for secondary objective 4 (1B.SO4) will descriptively compare the restricted variable FMF screening test to the history-based risk factor only screening tests (NICE/ACOG/other international agencies/current standard of care in Ghana, Kenya and South Africa) using receiver operating characteristic (ROC) curves, the area under the ROC curves (AUCs) (C-statistic), over suitable risk threshold intervals (e.g. 0.1 percentage points, as needed). Calibration plots will also be compared.

Analysis for secondary objective 5 (1B.SO5) will estimate the relevant population-level binomial proportions (for binary data, e.g. referral to high-level care, use of aspirin, use of calcium supplement), medians (for count data, e.g. number of antenatal visits and gestational age at first antenatal clinic visit) with the method used to depend on the distribution of the count data, with 95% confidence intervals. The aspirin coverage in the study population is currently unknown, however, we expect the coverage to be low. If the aspirin coverage is high, this will affect the primary outcome, and it may have impact on the prognostic performance of the restricted FMF risk screening tool. Therefore, we will compare the characteristics of participants who received aspirin with those who did not and calculate the adjusted proportions of the primary outcome. Furthermore, we will estimate these outcomes amongst women who screen positive (i.e. high-risk women) and those who screen negative.

Maternal and fetal secondary endpoints collected as part of Objective 1B will be summarised descriptively using frequency and proportions. These data will be used to inform preparations for the PEARLS trial. These will be considered for any relevant scenario or definition for the eligible population for the randomised trial – for example if certain subgroups of women (multiples, history of stillbirth/FGR, etc) are automatically considered as high risk or not.

**Exploratory objective**

Analysis for exploratory objective 1 (1B.EO1): An alternative risk prediction model for delivery with pre-eclampsia before 37 weeks of gestation will be explored. This model may be developed using either a standard regression-based method or a machine learning based method. Internal validation of this model will be performed using 5-fold cross-validation and bootstrap validation. This may also include the use of other variables that are collected prospectively in the cohort study, but have not been included in FMF models previously (such as presence of sickle cell disease, HIV, TB, malaria and other co-morbidities). It may also include use of variables within the FMF model, but with a greater level of detail. For example, FMF model as included presence/absence of chronic hypertension, though PEARLS has data on presence/absence chronic hypertension, antihypertensive drug use, whether chronic hypertension is controlled or uncontrolled at time of screening, and whether chronic hypertension is diagnosed. We will compare the performance of this alternative risk prediction model to see if it is meaningfully better than the restricted-variable FMF approach.

For all analyses, data from the three countries will be pooled together. In addition, descriptive summary tables will be provided by country.

# 8 Sensitivity Analyses

No sensitivity analyses are planned.

# 9 Interim Analysis

An initial formal interim analysis will be conducted after approximately 67% of participants have been enrolled. Ideally, this is timed for when Ghana has completed recruitment of participants and at least 50% of participants recruited in Kenya and South Africa, though this may be approximate if recruitment rates are slower or faster than expected. This analysis will be for objective 1B, primary objective (1B.PO1), using only the screening/enrolment data to determine the threshold that gives a 10% positive screening rate (overall and by country).

A second formal interim analysis will be performed following completion of enrolment of 100% of planned participants, and all available data on pregnancy and delivery outcomes. We estimate that ~67% of participants will have delivered by this time, though this is approximate if recruitment rates are slower or faster than expected. This interim analysis will be for objectives 1B (including 1B.PO1, 1B.SO1, 1B.SO3, 1B.S04, 1B.S05). The results of the interim analyses will be shared with the Trial Steering Committee, and the Data Safety Monitoring Board (DSMB) prior to starting the PEARLS trial.

# 10 QC Plans

All data will be reviewed and cleaned before the start of any analysis. Data discrepancies will be recorded and resolved prior to undertaking the analysis. Any proposed changes to the data (e.g., due to implausible values identified for some variables) will be discussed with the team to reach consensus. All changes will be documented with rationale provided.

# 11 Programming Plans and allocation of who will complete the statistical analyses

Analyses will be undertaken using Stata/R. All analyses outlined in this statistical analysis plan will be undertaken by the University of Melbourne Methods and Implementation Support for Clinical and Health (MISCH) research Hub biostatistical team.

# 12 References

1 Collins, G. S., Reitsma, J. B., Altman, D. G. & Moons, K. G. M. Transparent reporting of a multivariable prediction model for individual prognosis or diagnosis (TRIPOD): the TRIPOD statement. *BMJ : British Medical Journal* **350**, g7594 (2015).

2 Tan, M. Y. *et al.* Screening for pre-eclampsia by maternal factors and biomarkers at 11-13 weeks' gestation. *Ultrasound Obstet Gynecol* **52**, 186-195 (2018).

3 Wright, D. *et al.* Predictive performance of the competing risk model in screening for preeclampsia. *Am J Obstet Gynecol* **220**, 199.e191-199.e113 (2019).

4 O'Gorman, N. *et al.* Competing risks model in screening for preeclampsia by maternal factors and biomarkers at 11-13 weeks gestation. *Am J Obstet Gynecol* **214**, 103.e101-103.e112 (2016).

5 Snell, K. I. E. *et al.* External validation of prognostic models predicting pre-eclampsia: individual participant data meta-analysis. *BMC Medicine* **18**, 302 (2020).

# 13 Appendix

**Table 1: Risk factors used for the prediction of pre-eclampsia**

| **Agency** | **Who to screen** | **Definition of high risk** | **Factors used in screening** |
| --- | --- | --- | --- |
| NICE (2019) | all pregnant women 11-13+6 | High: at least 1 high risk factor, or 2 moderate risk factors | History-based risk factors only*  *High risk factors:*   - History of HDP - Chronic hypertension - Type I or II diabetes - Renal disease - Autoimmune disease (systemic lupus erythematosus, antiphospholipid syndrome)   *Moderate risk factors:*   - Nulliparity - Obesity (BMI =/>35) - Family history of PET - Age 40 or over - +10 year pregnancy interval - Multiple pregnancy |
| ACOG (2020)  USPSTF (2021) | Pregnant women without hypertension or pre-eclampsia | High: one or more high risk-factors, or several moderate risk factors | History-based risk factors only*  *High risk factors:*   - History of PET, especially when accompanied by adverse outcome - Multifetal gestation - Chronic hypertension - Type I or II diabetes - Renal disease - Autoimmune disease (systemic lupus erythematosus, antiphospholipid syndrome)   *Moderate risk factors:*   - Nulliparity - Obesity (BMI >30) - Family history of PET (mother or sister) - Sociodemographic characteristics (African American race, low SES) - Age 35 or over - Personal history factors (LBW, SGA, previous adverse pregnancy outcome, +10 year pregnancy interval) |
| ISSHP (2018) | Pregnant women | Increased risk (depending on certain risk factors)  Clinical risk factors:  High: at least 1 high risk factor, or 2 moderate risk factors | History-based risk factors  *High risk factors:*   - History of PET - Obesity (BMI >30) - Chronic hypertension - Type I or II diabetes - Renal disease - Autoimmune disease (systemic lupus erythematosus, antiphospholipid syndrome) - IVF   *Moderate risk factors:*   - Nulliparity - Multifetal gestation - Maternal age >40 - Prior placental abruption - Prior stillbirth - Prior FGR   *Where integrated into local health systems:* MAP, UTPI, PLGF |
| WHO (2021) | Pregnant women | Moderate to high risk (depending on certain risk factors)  High: at least 1 high risk factor, or 2 moderate risk factors | No screening recommendation, but has a “non-exhaustive” list of risk factors  *High risk factors:*   - History of PET - Previous fetal or neonatal death associated with PET - Chronic or gestational hypertension - Diabetes - Renal disease - Autoimmune disease - Positive uterine artery doppler   *Moderate risk factors:*   - Primiparity - Family history of PET - Multifetal gestation - Maternal age >40 |
| Fetal Medicine Foundation | All pregnant women at 11-13+6 weeks | Low (<1 in 100)  High (>1 in 100) | History-based risk factors;   - Multifetal gestation - Maternal height - Maternal weight - Racial origin - Smoking during pregnancy - Family history of PET (mother) - Conception method - Chronic hypertension - Type I diabetes - Type II diabetes - systemic lupus erythematosus, - antiphospholipid syndrome - Nulliparous/Parous   MAP; UTPI; PLGF  PAPP-A (where PLGF not available) |
| FIGO (2019) | All pregnant women before the end of the first trimester | Low (<1 in 100)  High (>1 in 100) | *Ideal:* History-based risk factors (as per FMF); MAP; UTPI; PLGF  *Pragmatic:* History-based risk factors; MAP |
| Ghana  Guideline: Protocol for the Management of Hypertensive Disorders in Pregnancy | Not specified | \| Women are considered at risk if they have at least 1 high risk factor or at least 2 moderate risk factors \| \| --- \| | High risk factors:  - Hypertensive disease during a previous pregnancy  - Chronic hypertension  - Chronic Kidney Disease  - Autoimmune disease such as systemic lupus erythematosus or antiphospholipid syndrome  - Type 1 and 2 Diabetes  Moderate risk factors:  - First pregnancy  - Age 40 years or older  - Pregnancy interval of more than 10 years  - Body Mass Index (BMI) of 35 kg/m2 or more at first visit  - Family history of preeclampsia  - Multiple pregnancy |
| Kenya  Guideline: National Guidelines on Quality Obstetrics and Perinatal Care (February 2022) | First trimester | \| "Women are considered high risk if they have at least 1 history-based risk factor; low risk if they have no risk factors No guidance is provided on how to use tests/observations in the risk assessment" \| \| --- \| | *History based factors:*  - Family history  - Previous history of pre-eclampsia  - Chronic diseases such as renal disease, diabetes, hypertension  *Tests/observations:*  - Mean arterial pressure  - Biomarkers: placental growth factor, pregnancy associated plasma protein A  - Uterine artery pulsatility index  - Combination of these above risk assessment tests |
| South Africa  Hypertension in Pregnancy: National Guidelines (2018/19) | Not specified | Women are considered at risk if they have at least one risk factor. | *High risk factors:*  *-* Prior pre-eclampsia  - Chronic hypertension  - Multiple pregnancy  - Pre-gestational diabetes  - Maternal BMI >35  - Anti-phospholipid syndrome/SLE  - Assisted reproduction |
